# A survey of multidisciplinary healthcare providers utilizing the KNOWintegrativeoncology.org educational platform

**DOI:** 10.1186/s12906-022-03601-5

**Published:** 2022-04-28

**Authors:** Jen Green, Heather Wright, Dugald Seely, Mark Legacy, Maureen Anderson, Hallie Armstrong, Casey Martell, Sarah Soles, Lynda G. Balneaves

**Affiliations:** 1 Emcura Integrative Clinic, Bloomfield Twp, Bloomfield Hills, MI USA; 2 Goodapple Wellness, Philadelphia, PA USA; 3 Patterson Institute for Integrative Oncology Research, CCNM, Toronto, ON Canada; 4 The Centre for Health Innovation, Ottawa, ON Canada; 5 Beaumont Health Systems, Troy, MI USA; 6 Nayya Inc, New York, NY USA; 7 Integrated Health Clinic, Fort Langley, Langley, BC Canada; 8 College of Nursing, Rady Faculty of Health Sciences, University of Manitoba, 89 Curry Place, Winnipeg, MB R3T 2N2 Canada

**Keywords:** Herbs, Supplements, Cancer, Health information, Internet, Integrative oncology, Education, Comprehensive cancer care

## Abstract

**Background:**

Although the vast majority of cancer patients use natural health products (NHPs), 59% of oncology healthcare providers (HCP) report not receiving any education on NHPs. KNOWintegrativeoncology.org (KNOW) is a web-based educational platform that provides up-to-date evidence on NHPs used in cancer care with a user-friendly interface. KNOW is a database of human studies systematically gathered from MEDLINE and EMBASE. We surveyed HCPs before and after accessing KNOW to identify their information needs regarding NHPs in cancer care, their preferred way to receive information, barriers they face accessing NHP information, and to obtain feedback on the website.

**Methods:**

Recruitment was done through Beaumont Health Systems, the Society for Integrative Oncology, and the Andrew Weil Centre for Integrative Medicine, University of Arizona. HCPs who consented completed an initial survey and then a follow-up survey after being given access to KNOW for 4–6 weeks. Participants were required to access KNOW at least three times before completion of the follow-up survey.

**Results:**

A total of 65 participants completed the initial survey, with 60% (*n* = 39) from the conventional medical community, 33% (*n* = 21) from the integrative medicine community, and 7% (*n* = 5) from the research community. The majority of participants (82%; *n* = 53) preferred educational websites to email updates, podcasts/webinars, in-house experts, PubMed searches and smartphone apps. The most common barriers identified to accessing information on NHPs were time, accessibility at point-of-care, and credibility of sources. A high number of participants were lost to follow up, with 18 participants demographically representative of the initial sample of 65 completing the follow-up survey. Half (*n* = 9) of participants stated accessing the KNOW website changed their clinical practice. Close to 90% (*n* = 16) reported they would recommend KNOW to a colleague.

**Conclusion:**

Oncology HCPs reported preferring to use, and already relying on, numerous web-based educational platforms to gather information on NHPs, with time, accessibility, and credibility being common barriers to obtaining information. Our study findings highlight the promise of the KNOW web-based educational platform in reducing barriers to accessing up-to-date information on NHPs in busy cancer care settings.

**Supplementary Information:**

The online version contains supplementary material available at 10.1186/s12906-022-03601-5.

## Background

While the majority of cancer patients actively use natural health products (NHPs) during their systemic treatment for cancer [1–5], knowledge about the broader field of complementary and integrative medicine (CIM) amongst the conventional healthcare community is lacking. In a 2014 survey of American Society of Clinical Oncology members, two-thirds of oncologists indicated they did not have enough knowledge to answer questions from patients regarding herbs and supplements, and 59% had not received any education about the topic [6]. Oncology healthcare practitioners (HCPs) in Canada were surveyed in 2015 and 69% reported receiving no formal training regarding NHPs, indicating an urgent need for improved CIM therapy education [7]. A third survey completed in Australia revealed that general practice doctors perceived a high level of need for CIM information resources due to a lack of knowledge coupled with a substantive level of interest to learn more [8]. Lastly, oncology HCPs in Germany were surveyed on their needs and preferences regarding CIM information and training [9]. Respondents highly preferred lectures, information platforms on the internet, workshops, and e-mail newsletters as methods of receiving information about CIM, and wanted summarized information that would support their understanding of how CIM therapies might aid in the management of side effects that arise from common conventional cancer treatments (e.g., chemotherapy and radiation).

In 2019, a study conducted in the United States audio-recorded patient‐clinician interactions in medical oncology outpatient practices. Conversations that included discussions about CIM were found to have more psychosocial statements from both clinicians and patients, and were rated higher on patient‐centeredness, positive patient and clinician affect, and patient engagement [10]. It is unrealistic, however, to expect oncology HCPs to have productive discussions about CIM when they largely feel unprepared to do so [6]. Oncology HCPs urgently need and want to have easy access to evidence-based CIM information. Increasing HCPs’ knowledge about CIM therapies, particularly NHPs, can help ensure patient information needs about these therapies are met, thus allowing for safer and more comprehensive cancer care.

In 2015, the Oncology Association of Naturopathic Physicians (OncANP) established KNOWintegrativeoncology (KNOW) an up-to-date, searchable database of human studies on NHPs in cancer care. KNOW’s methodology involves systematically searching MEDLINE and EMBASE and then parallel screening these citations for inclusion criteria (English language, human oncology studies on secondary prevention: systematic reviews, meta-analyses, observational studies, clinical trials and case reports that include the use of herbs, supplements or diet). Included studies are then extracted for key information about population, intervention, comparator and outcome (PICO). For controlled trials, a Cochrane Assessment of Risk of Bias for Randomized Trials is conducted (11). The KNOW medical librarian, who had contributed to the building of the PubMed CIM filter, guided a multidisciplinary team of HCPs and researchers in creating a detailed custom search filter for KNOW that expanded on existing CIM filters. The custom search filter was built using naturopathic expertise to include synonymous terms and detailed keywords for herbs, supplements, nutrients and diet to capture more studies in MEDLINE and EMBASE.

In 2016, KNOW evolved from a research database to a web-based educational platform designed to support integrative oncology education and clinical decision making. The KNOW team gathered user feedback from the Oncology Association of Naturopathic Physicians (OncANP) membership (450 HCP’s) through an online survey as well as an hour-long interview of beta testers at various stages of their careers. Based on this feedback, the KNOW website was extensively revised to make data searchable by tumor type, conventional treatment type, treatment side effect (e.g., peripheral neuropathy, nausea and vomiting, bone marrow suppression) and natural therapy type (e.g., green tea, Mediterranean diet, omega 3 fatty acids).

The decision to include EMBASE in KNOW’s search strategy has proved to be important because international studies on NHPs identified in EMBASE are often lacking in PubMed/ MEDLINE search results. To demonstrate the reliability of KNOW’s search strategy compared to one conducted in PubMed, a quality analysis was undertaken in 2017 and again in 2019 by two independent reviewers. The results were presented at the Society for Integrative Oncology Conference and Oncology Association of Naturopathic Physicians conference in 2019 [11, 12]. Both of the quality analyses found significantly more title results in KNOW than in PubMed. For example, in a search for “Mucositis” + “cancer” + “glutamine”, a total of 38 relevant studies were identified through both PubMed and KNOW. The KNOW search strategy identified 32 of the 38 studies, with 18 studies not identified via PubMed. In contrast, the PubMed search identified 20 of the 38 studies, with 6 not identified by KNOW. This comparison highlights the importance of including EMBASE in KNOW’s search strategy to yield more robust results.

The KNOW platform is updated quarterly and contains studies published in EMBASE and MEDLINE from the year 2000 to present. Search results are listed according to level of evidence, with meta-analyses and systematic reviews listed first, then clinical trials, followed by observational studies and, finally, case reports. Operationally, the KNOW platform allows users to search for studies, copy and paste references, and link online to an article’s abstract or full-text. The KNOW web-based educational platform is utilized by such organizations as the University of Arizona’s Andrew Weil Center for Integrative Medicine, the Canadian College of Naturopathic Physicians, and the Society for Integrative Oncology, with an estimated reach of over 2,500 HCPs and researchers.

The aim of this research study was to describe oncology HCPs’ information needs regarding NHPs in cancer care, their preferred way to receive information, barriers they face accessing NHP information, and to obtain feedback from multidisciplinary providers on the KNOW web-based educational platform. In addition, the perceived impact of using KNOW on HCPs’ clinical practice was explored.

## Methods

### Study design

A pre-post survey design was utilized in this study. Participants were required to answer two surveys: one at baseline and another at 4–6 weeks after accessing and using the KNOW website on at least three occasions.

### Participants and setting

Participants were included in this study if they were oncology HCPs or integrative oncology researchers in the United States. Professions included were: medical oncologists, oncology nurses, oncology physician assistants, oncology pharmacists, radiation oncologists, oncology surgeons, oncology fellows, naturopathic physicians working in oncology and integrative oncology researchers. Enrollment began September 24^th^, 2020 and ended March 14^th^, 2021. Recruitment announcements were made via email through Beaumont Health Systems in Michigan, the Society for Integrative Oncology, and the Andrew Weil Center for Integrative Medicine at the University of Arizona. There was no direct cost or compensation to participate in the survey; however, participants were offered access to the website for 12 months after completing the study.

### Data collection

The initial survey was comprised 18 items that assessed HCPs’ demographics (profession and age), information-seeking behavior related to NHPs (including resources used), preferred sources of NHP information, barriers to accessing NHP information, perceived credibility of NHP information resources, and continuing education needs related to NHPs. HCPs’ knowledge about NHPs and preparedness to address clinical issues associated with NHPs were also assessed, but not a focus of this study (Supplementary Material 1). The follow-up survey included the same questions as the initial survey (minus demographics), with the addition of eight items that assessed how accessing and using KNOW affected HCPs’ clinical practice, what improvements were required to the KNOW platform, and whether HCPs would recommend KNOW to other clinicians (Supplementary Material 2). The surveys were created by a multidisciplinary research team composed of both conventional and CIM HCPs and researchers. Surveys were distributed to consenting participants using the Research Electronic Data Capture (REDCap) website.

### Statistical analysis

Descriptive statistics (i.e., frequency, means) were used in this analysis to summarize key study variables.

## Results

### Participants

In total, 65 HCPs consented to participate and completed the initial survey. Table 1 shows the sample demographics. A total of 60% (*n* = 39) of respondents were conventional HCPs, 33% (*n* = 21) were CIM HCPs, and 7% (*n* = 5) were integrative oncology researchers. Age distribution was diverse, with most respondents in the 40 to 49-years group. Only 18 respondents completed the follow-up survey after accessing KNOW, with profession and age distributions similar to that of the larger initial group surveyed at baseline.

**Table 1:** Sample demographics at baseline and follow-up

	Baseline (*n* = 65)	Follow-up (*n* = 18)
*Profession*		
Medical oncologist	22 (33.8)	6 (33.3)
Naturopathic physician	15 (23.1)	4 (22.2)
Nurse practitioner	6 (9.2)	1 (5.6)
Integrative medical doctor	5 (7.7)	2 (11.1)
Integrative oncology researcher	5 (7.7)	2 (11.1)
Radiation oncologist	3 (4.6)	**-**
Registered nurse	3 (4.6)	1 (5.6)
Pharmacist	2 (3.1)	1 (5.6)
Physician assistant	2 (3.1)	1 (5.6)
Oncology fellow	1 (1.5)	**-**
Other	1 (1.5)	**-**
*Age Group (Years)*		
20–29	3 (4.6)	1 (5.6)
30–39	10 (15.4)	3 (16.7)
40–49	26 (40.0)	5 (27.8)
50–59	17 (26.2)	6 (33.3)
60–69	7 (10.8)	3 (16.7)
70 +	2 (3.1)	1 (5.6)

### Obtaining information on natural health products

The frequency of accessing information on NHPs varied between conventional and integrative HCPs (Table 2). Amongst conventional HCPs, 44.7% (*n = *17) reported gathering information daily or weekly, while the other 55.3% reported doing so monthly or less than monthly. Amongst integrative HCPs, 95.2% (*n* = 20) reported seeking information daily or weekly, while only one person (4.8%) reported doing so monthly.

**Table 2:** Frequency of gathering information about NHPs at baseline

Baseline	Conventional HCPs (%)	CIM HCPs (%)	Researchers (%)
	*n* = 38^a^	*n* = 21	*n* = 5
Daily	5 (13.1)	13 (61.9)	-
Weekly	12 (31.6)	7 (33.3)	-
Monthly	9 (23.7)	1 (4.8)	2 (40.0)
Less than monthly	12 (31.6)	-	3 (60.0)

^a^Conventional provider missing *n* = 1

*Abbreviations*: *CIM* complementary and integrative medicine, *HCPs* health care providers, *NHPs* natural health products

Table 3 shows the ways through which HCPs reported accessing information on NHPs. A total of 86.2% (*n* = 56) of respondents stated they used a website to obtain information, with 78.5% (*n* = 51) reporting they specifically used PubMed to search for information. Other top websites included Memorial Sloan Kettering Cancer Center, UpToDate, the National Cancer Institute, Natural Medicines Research Collaboration, and Beyond Conventional Cancer Therapies (see Supplementary Table 1). Amongst the integrative HCP community, PubMed (95.2% integrative vs. 66.7% conventional) and conferences (71.4% integrative vs. 43.6% conventional) were more frequently used compared to within the conventional HCP community. Websites not including PubMed, in general, saw more usage in the conventional HCP community compared to the integrative HCP community (94.9% conventional vs. 76.2% integrative). All other methods were used approximately the same between groups.

**Table 3:** Frequency of actual NHP information resources used

Resources Used	Conventional HCPs (%)	CIM HCPs (%)	Research Staff (%)	TOTAL (%)
	(*n* = 39)	(*n* = 21)	(*n* = 5)	(*n* = 65)
Websites	37 (94.9)	16 (76.2)	3 (60.0)	56 (86.2)
PubMed Search	26 (66.7)	20 (95.2)	5 (100.0)	51 (78.5)
Conferences	17 (43.6)	15 (71.4)	2 (40.0)	34 (52.3)
Webinars or Podcasts	16 (41.0)	13 (61.9)	1 (20.0)	30 (46.2)
Google	15 (38.5)	9 (42.9)	3 (60.0)	27 (41.5)
In-House Expert	22 (56.4)	8 (38.1)	1 (20.0)	21 (32.3)
Email Updates	9 (23.1)	10 (47.6)	-	19 (29.2)
Smartphone Apps	6 (15.4)	3 (14.3)	1 (20.0)	10 (15.4)
E-Textbooks	3 (7.7)	6 (28.6)	-	9 (13.8)

*Abbreviations*: *CIM* complementary and integrative medicine, *NHP* natural health products

Table 4 shows the ways HCPs preferred to gather information on NHPs. Websites were the most preferred source (81.5%; *n* = 53). There were, however, large differences in actual vs. preferred means of obtaining information: PubMed (78.5% actual vs. 47.7% preferred), conferences (52.3% actual vs. 16.9% preferred) and Google (41.5% actual vs. 7.7% preferred). These discrepant results may indicate a reliance on non-preferred sources of information whereas HCPs would prefer websites for information about NHPs.

**Table 4:** Frequency of preferred NHP information resources

Preferred Resources	Conventional Providers (%)	CIM HCPs (%)	Research Staff (%)	TOTAL (%)
	(*n* = 39)	(*n* = 21)	(*n* = 5)	(*n* = 65)
Websites	34 (87.2)	17 (81.0)	2 (40.0)	53 (81.5)
Email Updates	20 (51.3)	14 (66.7)	3 (60.0)	37 (56.9)
Webinars/Podcasts	14 (35.9)	16 (76.2)	5 (100.0)	35 (53.8)
In-House Expert	22 (56.4)	8 (38.1)	2 (40.0)	32 (49.2)
PubMed Search	23 (59.0)	8 (38.1)	-	31 (47.7)
Smartphone Apps	15 (38.5)	2 (9.5)	-	17 (26.2)
Conferences	5 (12.8)	5 (23.8)	1 (20.0)	11 (16.9)
E-Textbooks	5 (12.8)	3 (14.3)	-	8 (12.3)
Google	2 (5.1)	3 (14.3)	-	5 (7.7)

*Abbreviation*: *CIM* complementary and integrative medicine, *NHP* natural health product

### Barriers to accessing information on NHPs

HCPs were asked to rate the barriers they experienced in obtaining information about NHPs on a scale of 0–5, where 0 is no barrier and 5 is a strong barrier. The strongest barriers according to mean intensity level were: time limitations (3.4/5), accessibility of data at point-of-care (3.1/5), credibility of sources (3.0/5), and lack of in-house experts to consult (2.2/5). Table 5 shows the full data on these four barriers. Other individual barriers identified included; lack of evidence, lack of detailed information on some products, cost and the organization and searchability of data.

**Table 5:** Barriers to accessing NHP information

Rating	Time (%)	Credibility of Sources (%)	Accessibility at POC	Lack of in-house expert
0 (No Barrier)	2 (3.1)	5 (7.7)	3 (4.6)	20 (30.8)
1	5 (7.7)	5 (7.7)	7 (10.8)	7 (10.8)
2	6 (9.2)	7 (10.8)	8 (12.3)	9 (13.8)
3	20 (30.8)	27 (41.5)	20 (30.8)	8 (12.3)
4	16 (24.6)	12 (18.5)	19 (29.2)	9 (13.8)
5 (Strong Barrier)	16 (24.6)	9 (13.8)	8 (12.3)	12 (18.5)
Mean	3.4/5	3.0/5	3.1/5	2.2/5

*n* = 65. *Abbreviation*: *POC* point-of-care, *NHP* natural health product

### Impact of accessing KNOW

Of the 18 post-survey respondents, 50% (*n* = 9) stated that accessing the KNOW platform changed their clinical practice**. **Of the nine HCPs who felt their practice had changed, 77.8% (*n* = 7) said that accessing the website made their clinical decision more evidence based, 44% (*n* = 4) felt more confident answering questions related to natural products, 44.4% (*n* = 4) had a greater understanding about herb/supplement-drug interactions, and 44.4% (*n* = 4) felt more comfortable recommending natural products they had not previously recommended.

### Recommended improvements to KNOW

Recommendations for improvements to the KNOW platform from the 18 post-survey respondents fell into several categories, the most common being technical (i.e., search engine optimization, user interface and development of a smartphone app), integration into other resources (i.e., existing web-based health resources, linking to other internet-based resources), and expansion of scope (i.e., new topics, herb-drug interactions). Three-quarters of HCPs noted they would like to “add clinical tips” to the KNOW website and 38% suggested the KNOW developers “create an app”. Finally, 12.5% wanted additional information, such as NHP dosing and interaction with drugs (with links to supporting studies), and the mechanism of action of select NHPs.

With regards to the format in which studies are summarized, over 50% of respondents said they would accept a simple language summary of each research study, with a hyperlink to the study online. However, in the KNOW platform, features like full extractions of studies with details on the population, intervention, comparator and outcome (PICO), as well as the Cochrane quality/risk of bias assessment tool [13], were rated as important or very important by 75% of respondents.

### Satisfaction with KNOW

The majority of follow-up survey respondents (88.9%; *n* = 16) said they would recommend KNOW to a colleague or to their professional organization. Participants noted that the website was practical, helped save time, was comprehensive, and that it helped fill an unmet need as a resource for evidence-based integrative oncology recommendations. One respondent noted they did not think that it was more useful than PubMed. All qualitative feedback can be found in the Supplementary Material 3.

## Discussion

This survey is consistent with previous surveys that show variability in how often oncology HCPs gather information about NHPs [7] and that their preferred information source is web-based platforms [9]. In our study, integrative HCPs reported gathering information more frequently and consistently compared to conventional HCPs, with almost all integrative HCPs collecting data at least weekly. Conventional providers, in contrast, varied more in how often they accessed information, with over 50% reporting monthly or less than monthly access. There was similarity in the ways that all HCPs access information, as well as the ways they prefer to access information, with websites being the top choice in each instance. It was interesting to note that there was great variability in the specific websites used, with five separate websites being used by more than 50% of respondents. This implies that HCPs use multiple websites in sourcing information about NHPs. This practice may be beneficial for minimizing the effects of bias that can occur from relying on any single website. It also suggests that one website alone was not sufficient to address questions about NHPs.

The most substantial barrier to accessing information was time, with 80% of respondents rating it 3 or greater out of 5. The second highest rated barrier to accessing NHP information was accessibility at point-of-care. Considering these barriers, it is reasonable that HCPs prefer web-based platforms to email updates (which may be more time efficient but not as searchable at point-of-care), podcasts/webinars (time consuming and not searchable at point-of-care), in-house experts (time efficient but not accessible in most cancer care settings), and PubMed searches (highly searchable at point-of-care but not time efficient). There was also a discrepancy of 30% or more between actual use versus preferred use of PubMed, conferences, and Google, perhaps indicating that there is a need to rely on these non-preferred information sources. In a previous survey, HCPs reported they were frequently unable to locate the CIM information they needed, had limited knowledge of existing CIM resources, and instead relied on MEDLINE [14]. Searchable educational websites, available at point-of-care, are likely the best way to assist HCPs in accessing information about NHPs.

Credibility of resources was rated, on average, 3 out of 5 as a barrier to accessing NHP information. In a previous study on web-based information about herbal medicines used in cancer care, it was found that most sites were low quality on a number of indicators, including accuracy of information, revealing sources of information, biased presentation of information or regularity of updates [15]. A number of features in KNOW help to avoid low quality indicators and augment its credibility, including conducting regular searches of EMBASE and MEDLINE and displaying the search results according to level of evidence. Full summaries provide an extraction of human research studies in chart format that includes key indicators informative to clinical decision making, such as the tumor type and conventional treatment plan, the dose and route of administration of NHPs, observed side effects, adverse events and interactions, and details regarding statistical analyses (i.e., confidence intervals and p-values). A potential weakness of KNOW is that while side effects are noted, general cautions regarding NHPs are not included in the platform. This means that HCPs without background knowledge in NHPs may need to consult additional websites to learn more about safety considerations through monographs on specific NHPs. A possible improvement to KNOW would be to link to well referenced monographs, such as the National Cancer Institute’s Physician Data Query Cancer Information Summaries for Integrative, Alternative, and Complementary Therapies.

Limited time, which was a significant barrier for HCPs seeking NHP information, may also account for why only 18 of the 65 initial respondents completed the follow-up survey. Oncology HCPs are increasingly burdened with growing caseloads, care complexity, and administrative burdens [16]. Other possible reasons for the low completion rate of the follow-up survey include its timing during the COVID-19 pandemic, lack of financial incentive, technical challenges accessing the KNOW website, and failure to use the KNOW website at least three times.

Despite the low final survey response rate, those who did respond came from diverse healthcare professions that reflected the demographics of the larger group of initial respondents. Final respondents were highly satisfied with the KNOW website. Most respondents identified search engine optimization and user interface as areas for improvement. There was contradictory feedback with regards to the summarizing format. Within KNOW, randomized controlled trials have a full summary that includes PICO, plus a Cochrane quality analysis. In contrast, most other study designs (systematic reviews, observational studies, case reports) contain just a summary with a link to the study and back end tagging of keywords to make it more searchable. Final respondents simultaneously indicated that a full PICO/Cochrane summary was important to them, but they would also appreciate a simple summary format. Future surveys will need to be conducted to confirm which style of study summary HCPs prefer and to gain input from a larger group of oncology HCPs.

The main limitation of this survey is the small group surveyed and low proportion of the group that provided feedback after utilization of the KNOWintegrativeoncology.org tool. There was a selection bias in favor of HCPs who have an affinity for such a tool because others who were less likely to use such an information source would likely have decided not to access KNOWintegrativeoncology.org in the first place and, thus, not completed the study. To address this limitation and expand on the generalizability of our findings, further survey work in another setting will be important to conduct.

## Conclusions

This study gathered information about conventional and CIM HCPs' information needs regarding NHPs in their daily oncology practices. Most of the HCPs in the study preferred to use, and already relied upon, web-based educational platforms to gather information; however, there were significant barriers to accessing information about NHPs, including time, accessibility at point-of-care, and credibility of the information. One potential solution is the development of a comprehensive website that contains up-to-date and reliable information about NHPs that is easy to navigate and can be used directly at the point-of-care. The goals of the KNOW platform are in line with these needs and holds potential for application in busy cancer care settings. The supportive qualitative responses from some study participants regarding the impact of KNOW on their clinical practice and their willingness to recommend the website to colleagues highlight the promise of the KNOW web-based educational platform in reducing barriers to accessing NHP information. Future work will expand the survey to a larger sample within different locations within Canada and the United States to better assess the impact of the KNOW platform on oncology HCPs’ information needs and clinical practice related to NHPs.

## Supplementary Information

**Additional file 1:** **Supplementary 1:** Baseline Survey. **Supplementary2:** Follow-Up Survey. **Supplementary3:** Qualitative Feedback from open ended questions. **Supplementary Table 1.** Websites commonly used by Oncology Healthcare Providers.

## Data Availability

Due to privacy concerns and the cost of archiving data, the data for this study has not been deposited in a public repository. However, a de-identified copy of the dataset is available, on request, by contacting Jen Green (jengreennd@gmail.com).

## Abbreviations

CIM: Complementary and Integrative Medicine
KNOW: Knowledge in Integrative Oncology Website
NHP: Natural Health Product
OncANP: Oncology Association of Naturopathic Physicians
PICO: Population, Intervention, Comparison, and Outcomes
RCT: Randomized Controlled Trial
REDCap: Research Electronic Data Capture
