# Supplementary material for: A survey of multidisciplinary healthcare providers utilizing the KNOWintegrativeoncology.org educational platform
Source: BMC Complement Med Ther. 2022 Apr 28;22:118. doi: 10.1186/s12906-022-03601-5 (PMC9047465; doi:10.1186/s12906-022-03601-5)
Supplement: Supplementary file 1 — Additional file 1: Supplementary 1: Baseline Survey. Supplementary2: Follow-Up Survey. Supplementary3: Qualitative Feedback from open ended questions. Supplementary Table 1. Websites commonly used by Oncology Healthcare Providers. [file 12906_2022_3601_MOESM1_ESM.docx]

Supplementary Materials

Supplementary 1: Baseline Survey

“Natural products” in the survey below refers to vitamins (e.g. vitamin D), minerals (e.g. zinc), herbal remedies (e.g., green tea, turmeric), amino acids (e.g., glutamine, carnitine) and supplements (e.g., omega 3 fatty acids, probiotics, melatonin).

1. What type of oncology health professional are you?

- Medical oncologist
- Registered oncology nurse
- Oncology pharmacist
- Radiation oncologist
- Oncology surgeon
- Cancer researcher
- Oncology fellow
- Naturopathic physician
- Other (we ask for only cancer professionals to complete this survey): ______________________

2. What age group best captures your current age?

1. 20-29 years
2. 30-39 years
3. 40-49 years
4. 50-59 years
5. 60-69 years
6. 70+ years

3. Please rate your knowledge level for each of the following: (1 = Not at all knowledgeable, 2 = Somewhat knowledgeable, 3 = Knowledgeable, 4 = Very knowledgeable;

- Potential role of natural products in cancer treatment
- Potential role of natural products in cancer symptom management
- Potential role of natural products in cancer  survivorship
- Evidence to support safety/efficacy with use of natural products
- Risks and benefits of natural products in cancer care
- Natural product- drug interactions
- New/latest studies on natural products in cancer care

4. How prepared do you feel to: (1 = Not at all prepared, 2 = Somewhat prepared, 3 = Prepared, 4 = Very prepared)2   3   4/very prepared checkbox/want info)

- Assess patients’ natural product use
- Respond to patients’ requests for information about natural products
- Guide patients on possible negative interactions between natural products and conventional cancer care
- Help patients integrate natural products into their overall care
- Help patients identify and monitor for adverse events associated with natural product use
- Find credible information resources about natural products (e.g., websites, books, journal articles)
- Evaluate the credibility of natural product information resources
- Search for evidence about a natural product

5. How often do you gather information on natural products for cancer patients in your professional life? (1 = never, 2 = less than monthly, 3 = monthly, 4 = weekly, 5 = daily)

6. What resources do you currently use to answer patient questions on natural products (check all that apply):

- Websites
- Smartphone apps
- In-house expert
- PubMed search
- Conferences
- Online webinars/podcasts
- Email updates
- Google or google scholar
- E-textbooks

7. Please identify any websites or apps you currently consult for information on natural products in cancer care:

- UpToDate
- Natural Medicines Research Collaboration
- Memorial Sloan Kettering Cancer Center’s About Herbs
- National Center for Complementary and Integrative Health (NCCIH)
- CAM-Cancer
- National Cancer Institute (NCI)/Office of Cancer Complementary Alternative Medicine
- Beyond Conventional Cancer Therapies
- Other:_____________________

8. How do you prefer to receive information on natural products?

- Websites/online educational platform
- Smartphone apps
- Webinar lectures on specific natural product-related topics
- E-mail newsletters
- In-house experts
- Publications in scientific journals/PubMed search
- Conferences
- Information brochures
- E-textbooks
- Other (please specify):______________________

9. Please rate the following potential barriers to you accessing information on natural products and cancer care on a scale of 0-5, where 0 =no barrier and 5 = strong barrier;

- Time
- Credibility of sources
- Accessibility of data at point of clinical care
- Lack of in-house expert to consult
- Other (please specify):___________

10. Do you think there are evidence-based natural products that can be useful in side effect management during cancer care? Y/N

11. Do you think that there are some natural products that can interfere with surgery, radiation, chemotherapy, targeted agents or immunotherapy? Y/N

12. Do you think there are any evidence-based natural products that can improve quality of life in cancer survivorship? Y/N

13. Do you think there are any evidence-based natural products that reduce cancer recurrence risk? Y/N

14. How much do you trust publications from different parts of the world on a scale of 1 to 5, where 1 is not trustworthy and 5 is very trustworthy?

- US      1   2  3  4  5
- Canada     1   2  3  4  5
- China     1   2  3  4  5
- Southeast Asia (e.g., India, Indonesia)   1   2  3  4  5
- Australia     1   2  3  4  5
- Western Europe     1   2  3  4  5
- Eastern Europe and Russia     1   2  3  4  5
- Central America     1   2  3  4  5
- South America     1   2  3  4  5

15. What natural products do you get the most questions about from patients?_________

16. For continuing education, what natural products are you most interested in learning more about?_________

17. For continuing education, what cancer treatment side effects are you most interested in learning more about?_________

18. General comments you would like to make about natural products and cancer care or KNOWoncology.org:_____________________________________________________________________________________________________________________________________________________________________________________________________________________________________________

Supplementary 2: Follow-Up Survey

These questions were added in the second survey in addition to all baseline questions.

1. Has your clinical practice changed since you accessed KNOWoncology.org? Y/N
2. If yes, in what ways? Click all that apply:
   - I feel more confident answering questions from patients related to natural products in cancer care
   - My clinical decision making around natural products in cancer care is more evidence-based
   - I have a greater understanding about herb-supplement-drug interactions
   - I feel more comfortable recommending natural products I didn’t recommend before
   - Other (please specify): ________________________
3. What improvements do you recommend for KNOWoncology?
   - Change user interface
   - Improve search engine
   - Edit summaries more carefully
   - Change the summary composition (explain - open text box)__________
   - Add clinical tips
   - Add features (open text box)______________
   - Create an app
   - Integrate into existing information resources eg. EPIC, UpToDate, Natural Medicine Research Collaboration
   - link out to additional internet based resources
   - Other______________________
4. If KNOWoncology were to change how it summarized studies, what would be enough data to meet your information needs?
   - Just a citation with link to PubMed abstract/EMBASE abstract
   - 2-3 sentence simple language summary with link to study
   - Full summary with details on PICO/population, intervention, study design and outcomes
   - Other (please specify): _______________________________
5. How valuable are the curated abstracts/drop down PICO summaries to you? (1 = Not at all important, 2 = Somewhat important, 3 = Important, 4 = Very Important)
6. How valuable was the Cochrane quality/risk assessment tool included in some of the summaries? (1= Not at all important, 2 = Somewhat important, 3 = Important, 4 = Very Important)
7. Would you recommend KNOWoncology.org to a colleague? Y/N  Why/why not?_____________
8. Would you recommend KNOWoncology.org to your professional organization? Y/N Why/why not?_________________________

Supplementary 3: Qualitative Feedback from open ended questions

“*Why would you recommend KNOW to a colleague or why not*?”;

“Not better than Pubmed” ”Very helpful for practice and patient management” “It is a useful tool that can help save time and improve quality of care” “We need more evidence-based resources in integrative oncology.” “resource that many do not know of” “It's a comprehensive tool to help guide evidence based integrative oncology recommendations” “reliability” “It is a useful tool that can help save time and improve quality of care” “I would recommend this to my organization so we have a database to link integrative oncology to our traditional practice.” “helpful” “great resource” “Loved the summaries, evidence based”

“*Why would you recommend KNOW to a professional organization or why not?*”

“I didn't find it more helpful than Pubmed” “I would recommend this to my organization so we have a database to link integrative oncology to our traditional practice." “It is very informational and educational, and is easy to use.” “I am interested in Integrative Oncology and found KNOW to be a good go to website” “A credible resource is always worthwhile sharing” “great resource” “reliability” “It's a comprehensive tool to help guide evidence based integrative oncology recommendations” “It brings a lot of useful information together into one place. Unlike a pub med search, you do not need to scroll through hundreds of studies to find what you need.” “I will once the Interactions tab is functional. I would use this tab to help determine if any natural products have been reported to interact with conventional drugs my company manufactures, any same-class drugs, or traditional chemotherapies we may use as comparators in our clinical trials.” “Again it is a great resource to augment the other available natural supplement websites.” “We need more evidence-based resources in integrative oncology.”

Supplementary Table 1 – Websites commonly used by Oncology Healthcare Providers

|  | Conventional Providers  N = 39 | Integrative/CAM Providers  N = 21 | Research Staff  N = 5 | TOTAL  N = 65 |
| --- | --- | --- | --- | --- |
| Memorial Sloan Kettering Cancer Center | 31 | 11 | 4 | 71% (46) |
| Up To Date | 23 | 12 | 1 | 55% (36) |
| National Cancer Institute | 21 | 10 | 4 | 54% (35) |
| National Center for Complementary and Integrative Health | 20 | 11 | 4 | 54% (35) |
| Natural Medicines Research Collaboration | 19 | 13 | 1 | 51% (33) |
| Beyond Conventional Cancer Therapies | 8 | 6 | 1 | 23% (15) |
| Cam-Cancer | 2 | 6 | 2 | 15% (10) |
| Consumer Lab | 2 | 1 | 0 | 5% (3) |
| Andrew Weil/ U Arizona | 2 | 1 | 0 | 5% (3) |
| Other | 2 | 3 | 0 | 8% (3) |

Numbers represent n of participants who reported to use the specific website.
